# Synthesis of a Temperature-Sensitive Matrine-Imprinted Polymer and Its Potential Application for the Selective Extraction of Matrine from Radix Sophorae Tonkinensis

**DOI:** 10.3390/ijms16023441

**Published:** 2015-02-04

**Authors:** Minjie Jiang, Lisheng Wang, Xu Liu, Hua Yang, Fan Ren, Lizhen Gan, Weizhe Jiang

**Affiliations:** 1 School of Chemistry and Chemical Engineering, Guangxi University, 100 Daxue Road, Nanning 530004, China; E-Mails: jmj247742311@gmail.com (M.J.); wendaoliuxu@163.com (X.L.); yanghua6316@sina.com (H.Y.); fancyren@163.com (F.R.); 2 School of Pharmaceutical, Guangxi Medical University, 22 Shuangyong Road, Nanning 530021, China; E-Mail: welldone.123@163.com

**Keywords:** molecularly imprinted polymer, *N*-isopropyl acrylamide, matrine, ultrasonic extraction

## Abstract

A temperature-sensitive matrine-imprinted polymer was prepared in chloroform by free-radical cross-linking copolymerization of methacrylic acid at 60 °C in the presence of ethylene glycol dimethacrylate as the cross-linker, *N*-isopropyl acrylamide as the temperature-responsive monomer and matrine as the template molecule. Binding experiments and Scatchard analyses revealed that two classes of binding sites were formed on molecular imprinted polymer (MIP) at 50 °C. Additionally, the thermoresponsive MIP was tested for its application as a sorbent material for the selective separation of matrine from Chinese medicinal plant radix Sophorae tonkinensis. It was shown that the thermoresponsive MIP displayed different efficiency in clean-up and enrichments using the SPE protocol at different temperatures.

## 1. Introduction

Molecularly imprinted polymers (MIPs), which have selective recognition site(s) for a target compound or its structurally related analog(s), have been used as chromatographic media, such as LC stationary phases or adsorbents for solid-phase extraction (SPE) and solid-phase microextraction [1,2,3]. Cross-linked *N*-substituted polyacrylamides are among the most widely studied polymeric materials used for the molecular imprinting of biomolecules such as proteins and DNA [4,5]. These polymers continue to receive much attention in the field of controlled drug delivery [6,7] because they can undergo a temperature-controlled volume phase transition in aqueous solutions.

**Figure 1:**
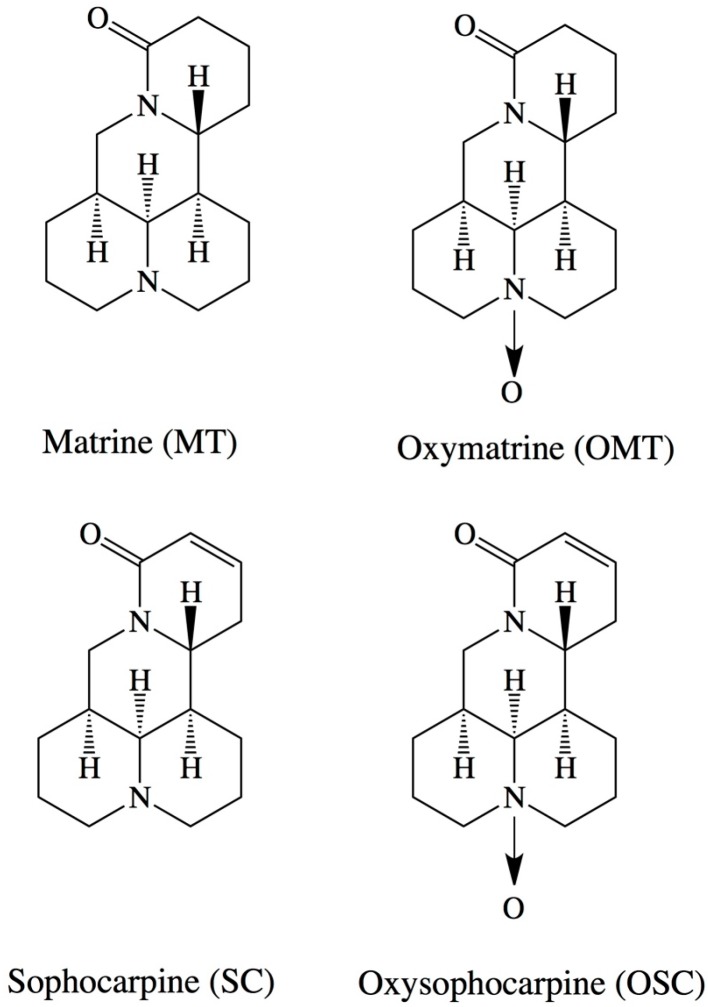
Chemical structures of matrine (MT), oxymatrine (OMT), sophocarpine (SC) and oxysophocarpine (OSC).

Radix Sophorae tonkinensis is a widely used traditional Chinese herbal drug that possesses strong biological activity. Matrine (MT, Figure 1) plays an important role in the treatment of aches, tussis, tumors and arrhythmia and serves as an antifebrile, diuretic and antidote [8,9]. Few studies have reported on the separation of MT via a molecular imprinting technique [10,11,12,13]. Therefore, it is important to develop efficient methods for the separation and determination of MT from Chinese herbs.

So far, to the best of our knowledge, few have reported in literature the method in thermoresponsive MIP for selective extraction of MT with water as a solvent. The aims of this study were to prepare thermoresponsive imprinted polymers using ethylene glycol dimethacrylate (EDMA) as a cross-linking monomer and *N*-isopropyl acrylamide (NIPA) as a temperature-responsive monomer and to compare the polymers’recognition ability to that of various structurally rigid polymers. The temperature dependence of the recognition ability of the prepared thermoresponsive polymers and the application of the polymers as an adsorption phase coupled with the ultrasonic for the selective extraction of MT from radix Sophorae tonkinensis were also investigated.

## 2. Results and Discussion

### 2.1. Polymer Synthesis and Characterization

The MT molecule has two hydrogen bonding sites, one on the amide group and another on the N atom of the heterocyclic group. The proper ratio of functional monomer to template molecules allows for a high adsorption capacity of the MIPs, whereas an excess of functional monomer enhances nonspecific adsorption. In the present work, we used a 1.5:4 ratio of template molecules to functional monomer as previously reported [11], and the binding of MT to thermosensitive imprinted polymers and structurally rigid polymers was compared. Normally, EDMA generates an imprinted polymer that is compact, inert and highly stable with respect to the rigidity of the polymer structure. Molecularly imprinted polymers and corresponding non-imprinted polymers, featuring NIPA and/or EDMA as a cross-linker, were created following a common protocol for MIP synthesis using the compositions listed in Table 1. The physical characteristics of the polymers were examined, and the results are summarized in Table 2. The results show that the pore diameter, pore volume and specific surface area of the NIPs and MIPs were not significantly different. When an excessive amount of NIPA was used during the pre-experiment stage, the polymers with soft texture and cannot be effectively reused were observed.

**Table 1:** Polymer composition. MIP: Molecularly imprinted polymer; NIP: Non-imprinted polymer.

Composition (mmol)	MIP1	NIP1	MIP2	NIP2	MIP3	NIP3	MIP4	NIP4
**Matrine (MT)**	1.5	0	1.5	0	1.5	0	1.5	0
**Methacrylic acid (MAA)**	4	4	4	4	4	4	4	4
**Ethylene glycol dimethacrylate (EDMA)**	9.6	9.6	14.4	14.4	19.2	19.2	28.8	28.8
***N*-isopropyl acrylamide (NIPA)**	19.2	19.2	14.4	14.4	9.6	9.6	0	0
**2,2′-Azobis(2-isobutyronitrile) (AIBN)**	1.9	1.9	1.9	1.9	1.9	1.9	1.9	1.9

**Table 2:** Characteristics of the synthesized polymers.

Polymer	Particle Size ^a^ (μm)	Pore Diameter (nm)	Pore Volume (μL/g)	BET Surface Area (m^2^/g)
**MIP1**	5.52 ± 0.03	21.19 ± 0.32	2.73 ± 0.18	7.42 ± 0.32
**NIP1**	6.01 ± 0.04	19.48 ± 0.29	1.91 ± 0.11	6.35 ± 0.28
**MIP2**	5.62 ± 0.03	18.25 ± 0.24	1.53 ± 0.09	6.28 ± 0.31
**NIP2**	6.33 ± 0.05	17.89 ± 0.23	1.65 ± 0.10	5.70 ± 0.22
**MIP3**	7.72 ± 0.06	13.32 ± 0.27	1.27 ± 0.08	4.41 ± 0.19
**NIP3**	9.37 ± 0.08	11.89 ± 0.17	1.19 ± 0.09	2.92 ± 0.12
**MIP4**	8.23 ± 0.08	16.11 ± 0.19	1.02 ± 0.07	3.62 ± 0.14
**NIP4**	12.78 ± 0.14	14.45 ± 0.23	0.76 ± 0.04	2.18 ± 0.11

^a^ Approximate mean particle size.

### 2.2. Binding Experiments

**Figure 2:**
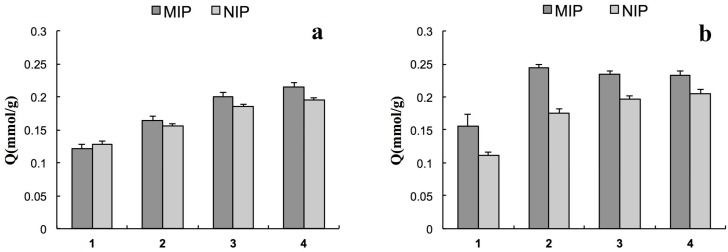
Binding of MT to molecular imprinted polymer (MIP) (1, 2, 3, and 4) and non-imprinted polymers (NIP) (1, 2, 3, and 4) at 25 °C (**a**) and 50 °C (**b**) (mean ± SD, *n* = 3).

**Figure 3:**
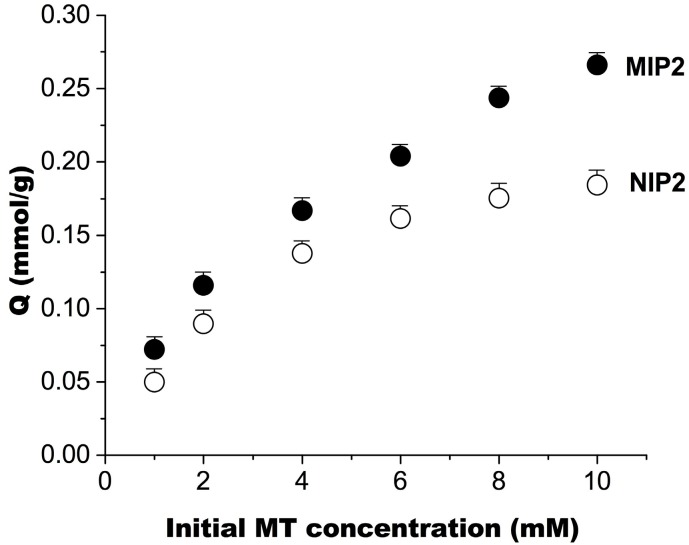
Binding isotherms of MT at 50 °C (mean ± SD, *n* = 3).

Parts a and b of Figure 2 show the binding of MT to MIP (1, 2, 3 and 4) and NIP (1, 2, 3 and 4) at 25 and 50 °C, respectively. At 25 °C, the amounts of MT bound to MIP gradually increased with the EDMA content and nothing different between MIPs and NIPs, but the amounts of MT bound to MIP were greater than the amounts of MT bound to NIP at 50 °C. Temperature-sensitive polymers are known for their ability to reversibly swell and shrink in response to environmental temperature changes. Due to such properties, the reversible and reproducible adsorption of target molecules was realized for the MIPs. In this study, MIP2 demonstrated the best adsorption capacity. Figure 3 shows the binding isotherms of MT on MIP2 and NIP2 at 50 °C; the binding amount increased gradually with an increase in MT concentration in the initial solution, but the amount of MT bound to MIP2 was greater than that bound to NIP2. Table 3 shows the *K*_d_ and Q_max_ values determined by Scatchard analysis. These data reveal that two types of binding sites, high- and low-affinity sites, formed on MIP2 at 50 °C, as previously reported [11], but at 25 °C, MIP2 and NIP2 exhibited similar properties.

**Table 3:** The binding characteristics of the thermoresponsive polymers at various temperatures. The correlation coefficients of the linear regression analyses were 0.955–0.999.

Temperature (°C)	MIP2				NIP2			
	High-Affinity Site		Low-Affinity Site		High-Affinity Site		Low-Affinity Site	
	*K*_d_ ± SE ^a^ (μM)	Q_max_ ± SE (μmol/g)	*K*_d_ ± SE (μM)	Q_max_ ± SE (μmol/g)	*K*_d_ ± SE (μM)	Q_max_ ± SE (μmol/g)	*K*_d_ ± SE (μM)	Q_max_ ± SE (μmol/g)
50	507 ± 63.5	197 ± 3.92	4210 ± 192	422 ± 66.3	–	–	1720 ± 277	225 ± 15.8
25	–	–	1620 ± 227	204 ± 24.5	–	–	1910 ± 223	202 ± 16.4

^a^ SE: standard error.

### 2.3. Molecular Recognition Properties of MIP2

**Figure 4:**
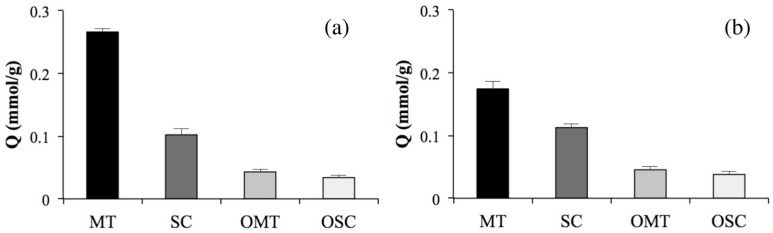
The specific adsorption of MIP2 at 50 °C (**a**) and 25 °C (**b**) (mean ± SD, *n* = 3).

Parts a and b of Figure 4 show the specific adsorption of MIP2 in a mixed solution of 10 mM MT, OMT, SC and OSC at 50 or 25 °C, respectively. MT and its structurally related compound, SC (13,14-dehydromatrine), were well recognized by MIP2, whereas neither OMT nor its structurally related compound, OSC (13,14-dehydrooxymatrine), were recognized at 50 °C. Additionally, the adsorption amounts of SC, OMT and OSC increased slightly at 25 °C, most likely due to polymer swelling. These results indicate that MIP2 can selectively recognize MT at 50 °C, but the recognition ability declines with decreasing temperature, which may be due to temperature-related swelling.

### 2.4. Temperature Effect on Recognition Ability

**Figure 5:**
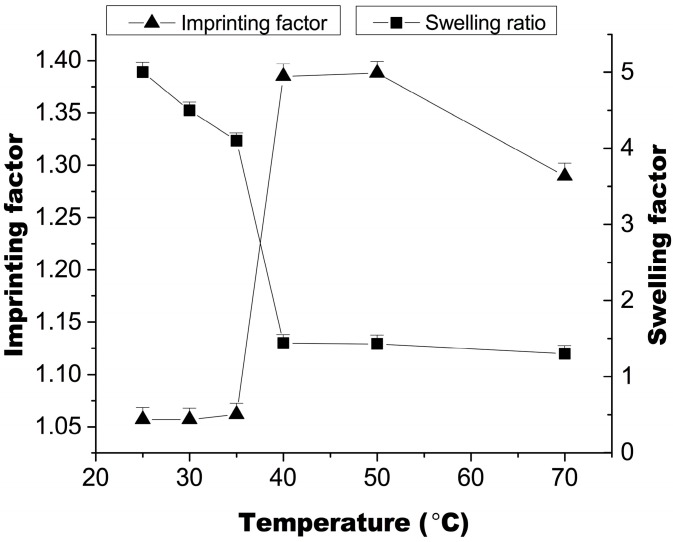
Binding affinity and swelling ratio of MIP2 (mean ± SD, *n* = 3).

The ability of the thermoresponsive polymer MIP2 to recognize a template molecule after a dynamic change in swelling was evaluated by equilibrium binding analysis. The experiment was performed at temperatures ranging from 25 to 70 °C. Figure 5 shows how the adsorption pattern of MT varied with temperature for MIP2, with sorption increasing as a function of temperature. The temperature-dependent swelling of the thermoresponsive MIP2 is also shown in Figure 5. As the temperature increased from 35 to 40 °C, it promoted binding of the template molecule to MIP2, whereas the corresponding NIP2 binding scarcely changed. The imprinting factor (the ratio of the amount of MT bound by the MIP to that bound by the corresponding NIP) of MIP2 was highest between 40 and 50 °C; this range appeared to correspond to the transition temperature of the thermoresponsive MIP, which was higher than that of the MIP prepared by precipitation polymerization [11]. At temperatures beyond 50 °C, it was observed that the increase in binding to the non-selective polymer was greater than the increase in binding to MIP, resulting in a decrease in the imprinting factor. The higher binding to the thermoresponsive MIP2 at high temperatures (>50 °C) is likely the primary consequence of an increase in the non-specific adsorption of the template molecules.

### 2.5. Recycling Times and Elution Process of MIP2

**Figure 6:**
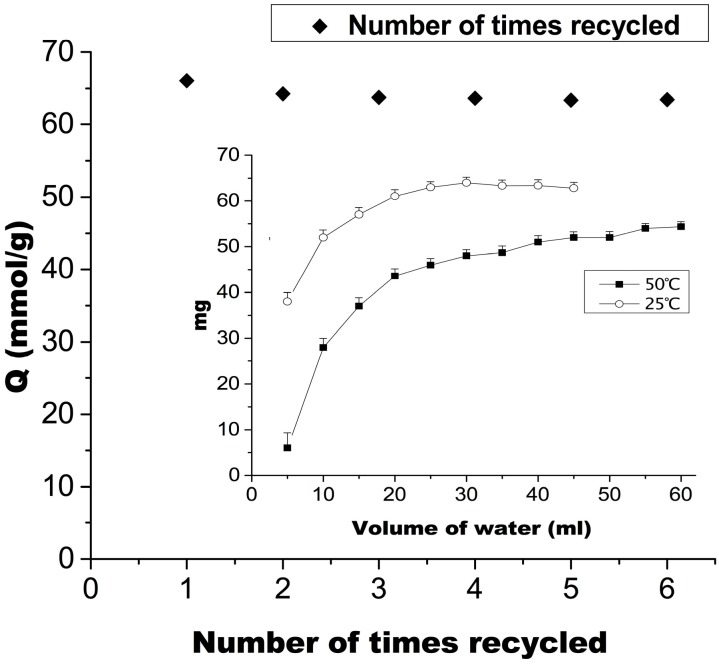
Number of times recycled and the relationship between elution volume and the quantity of MT extracted (mean ± SD, *n* = 3).

Figure 6 shows the relationship between elution volume and the amount of MT. These results indicate that water at 25 °C easily eluted MT from MIP2, whereas it showed lower efficacy at 50 °C. Thirty milliliters of water at 25 °C completely eluted MT. These results indicate that MIP2 can maintain the configuration required for the specific adsorption of MT at 50 °C. At 25 °C, when the polymer is swelled with water, the adsorption mechanism changes from being specific to being nonspecific, thereby facilitating the elution of MT. The adsorption capacity was not significantly reduced after six cycles of repeated use.

### 2.6. Selective Extraction of Matrine from Radix Sophorae Tonkinensis by MIP2 Coupled with Ultrasonic Agitation and Desorption

**Figure 7:**
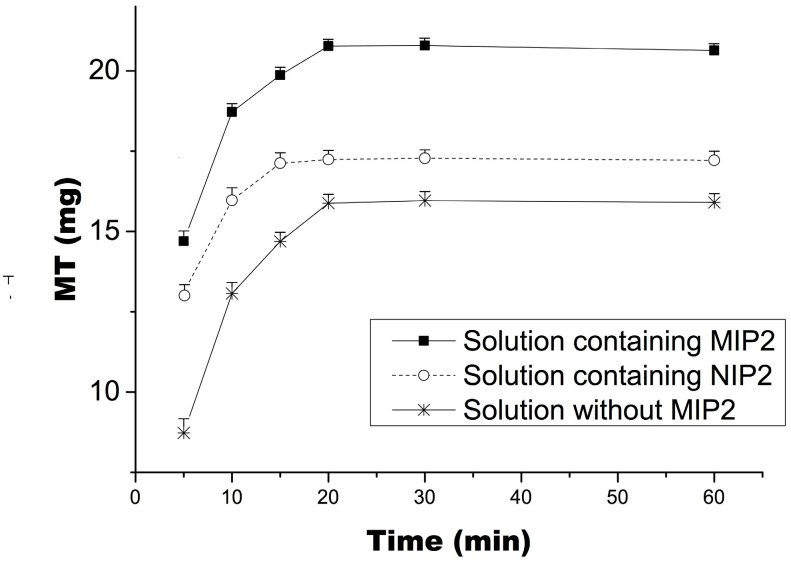
Total quantity of matrine (MT) extracted (mean ± SD, *n* = 3).

Figure 7 shows the total amounts of MT extracted as a function of time. “Solution without MIP2”, which represented the case in which MT was extracted from Radix Sophorae tonkinensis (before MIP2 extracted from radix Sophorae tonkinensis). On the contrary, “solution containing MIP2” which represented the case in which some MT was adsorbed onto MIP2 and some MT remained in solution (after MIP2 extracted from radix Sophorae tonkinensis, much higher than “solution containing NIP2”). These results suggest that MIP2 coupled with ultrasonic agitation can improve the dissolution rate of target compounds from Chinese medicinal herbs. Radix Sophorae tonkinensis contains approximately 2.43% matrine and oxymatrine (estimated by the Chinese Pharmacopoeia standard method).

**Figure 8:**
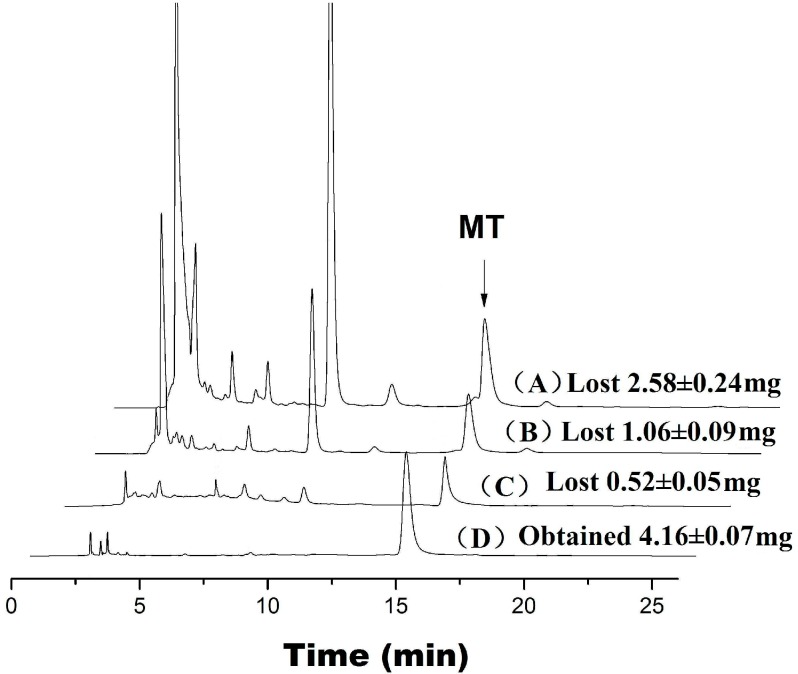
Desorption chromatograms of MIP2. A, B and C were eluted with 20, +10 and +10 mL of water with a temperature of 50 °C from saturated adsorption matrine samples; D were eluted with 30 mL of water with a temperature of 25 °C from **C** (mean ± SD, *n* = 3; Injection volume: 10 μL).

In order to evaluate the ability of the MIP2 for obtaining of high purity of MT, three samples of adsorbed MIP2 sample (20, 30 or 60 min) was eluted with 20, +10 and +10 mL of water (50 °C) for removing impurities at first; for the selective adsorption of MT, the eluted MIP2 sample was then eluted with 30 mL of water (25 °C). Figure 8 shows chromatograms of the desorption of MIP2. As indicated, some compounds were retained on MIP2 when eluted with 20 mL of 50 °C water (A), whereas using more 10 mL of 50 °C water resulted in the removal of nearly all impurities (B). Further more elution with 10 mL of 50 °C water removed all impurities in addition to a small amount of MT (C). Figure 8D shows the chromatograms for the MIPs eluted with 30 mL of 25 °C water from C. The results indicate that, after the removal of impurities with hot water, the cold water elution of the target compounds appears to be feasible.

## 3. Experimental Section

### 3.1. Chemicals and Materials

MT, OMT and OSC were purchased from the National Institute for the Control of Pharmaceutical and Biological Products (Beijing, China). SC was purchased from AvaChem Scientific (San Antonio, TX, USA). The chemical structures are shown in Figure 1. The Chinese herb radix Sophorae tonkinensis was purchased from a Chinese herbal drug store in Nanning, Guangxi Province, China. Methacrylic acid (MAA), *N*-isopropyl acrylamide (NIPA) and ethylene glycol dimethacrylate (EDMA) were purchased from Aldrich Chemical (Milwaukee, WI, USA). 2,2′-Azobis(2-isobutyronitrile) (AIBN) was supplied by the Special Chemical Reagent Factory of Nankai University (Tianjin, China). Methanol and chloroform were purchased from BDH Laboratory Supplies (Poole, UK). All solutions were prepared using high-purity water that had been purified by a Milli-Q plus system (Millipore, Molsheim, France).

### 3.2. Instrumentation

The liquid chromatography (LC) system used was composed of an LC-20AT pump, an SPD-20A UV-VIS Detector, an SIL-20A Autosampler, a CTO-20A Column Oven, and LC solution 20A (all from Shimadzu, Kyoto, Japan).

### 3.3. Polymer Synthesis

To obtain various structurally rigid polymers, four molecularly imprinted polymers (MIP1, MIP2, MIP3 and MIP4) and corresponding non-imprinted polymers (NIP1, NIP2, NIP3 and NIP4) were prepared using a thermal method involving free-radical polymerization according to previously reported methods [14]. To prepare these polymers, the polymerizing compositions listed in Table 1 were dissolved in 25 mL of chloroform. Subsequently, the polymeric mixtures were ultrasonically agitated under vacuum, purged with nitrogen for 5 min and polymerized by heating in a hot-air oven at 60 °C for 24 h. The resulting polymers were crushed, ground and sieved through a 200-mesh sieve. The print molecules were eluted from the polymer particles by washing with three 500 mL portions of 10% (*v*/*v*) acetic acid in methanol and subsequently with three 500 mL portions of methanol.

### 3.4. Characterization Methods

The mean size and the size distribution of the prepared particles were determined at 25 °C using laser diffraction (Malvern Mastersizer, Worcester, UK) with water as the suspending medium. The mean of triplicate measurements for the same batch was determined. The degree of polymer swelling was determined as the ratio of the volume of the swollen polymer to that of the dry polymer in water using a calibrated measuring cylinder. The pore volume and specific surface area were measured via nitrogen adsorption/desorption techniques using a Coulter SA3100 series surface area and pore analyzer (Beckman Coulter, Inc., Brea, CA, USA), which enables pores sizes between 0.3 and 200 nm to be measured. The samples were degassed at 120 °C, and a 50-point pressure table was used. The surface area was determined from a Brunauer, Emmett and Teller (BET) plot, whereas the average pore diameter and the cumulative pore volume were obtained using a Barrett, Johner and Halenda (BJH) model of the adsorption isotherm.

### 3.5. Binding Experiments

The ability of the prepared MIPs to selectively recognize the template molecule relative to the prepared NIPs was evaluated in water after equilibration of the polymers with an MT solution. In a typical binding assay, the powdered polymer (50 mg) of MIP or NIP was added to 5 mL of a water solution containing 8 mM of MT or 5 mL of the pure solvent (blank). After shaking at 25 or 50 °C for 10 h, an aliquot of each sample was taken and filtered through a 0.45 μm membrane filter. The concentration of free MT in the supernatant was determined by LC with an Inertsil ODS-SP, 5 μm column (250 mm × 4.6 mm I.D.); a column temperature of 40 °C; a mobile phase of 50 mM phosphoric acid (pH 2.3)-acetonitrile-methanol (940:50:10, *v*/*v*/*v*) featuring 160 mM sodium perchlorate; and a flow rate of 1.0 mL/min, with detection performed at 220 nm. The quantity of drug in solution was determined by referencing a calibration curve. The amount of bound drug was obtained by subtracting the amount of free drug from the total amount of drug added.

The binding of MT to the thermoresponsive imprinted polymer MIP2 and corresponding non-imprinted polymer NIP2 was examined at six different temperatures (25, 30, 35, 40, 45 and 70 °C) with a binding assay protocol. The specific adsorption of MIP2 was examined in a mixed solution of 10 mM of MT, OMT, SC and OSC at 25 or 50 °C. The imprinting factor (α), which represents the effect of the imprinting process, was the ratio of the amount of substrate bound by the MIP to that bound by the corresponding NIP.

### 3.6. Determination of the Thermoresponsive Polymers’ Binding Characteristics

The binding characteristics of a thermoresponsive imprinted polymer (MIP2) as well as the corresponding control polymer were examined at two different temperatures (25 and 50 °C) using 50 mg samples of polymer in MT solutions with concentrations ranging from 1 to 10 mM. The amount of MT bound (*Q*) was determined at each drug/polymer molar ratio (*R*). The binding parameters were determined from the equation Bound/Free = (*B*_max_ − *B*)/*K*_d_, where *K*_d_ is the equilibrium dissociation constant and *B*_max_ is the maximum number of binding sites, which were obtained from the slope and x-intercept of the corresponding Scatchard plot, respectively. The association constant (*K*_a_) was obtained as the reciprocal of *K*_d_. The mean MT binding constants were calculated based on results independently derived in triplicate.

### 3.7. Recycling Times and Elution Process of MIP2

Recycling time and elution experiments were performed using a glass SPE cartridge packed with 1 g MIP2 (MT adsorbed in 10 mmol/L MT solution for 10 h). A water elution temperature of 25 or 50 °C was used, after the aqueous solution flowed through the SPE cartridge under unforced conditions. The respective effluents were collected in a conical flask. The mean value was obtained from triplicate measurements of the same batch. Then, the relationships between the elution volume and the quantity of MT were calculated.

### 3.8. Selective MT Extraction from Radix Sophorae Tonkinensis by MIP2 Coupled with Ultrasonic Agitation and Desorption

Six samples were prepared via the following method. One gram of radix Sophorae tonkinensis was weighed and wrapped with 200-mesh nylon cloth and then extracted with 10 mL of water solution (containing 5% sodium bisulfite, which enables the formation of SC, OSC and OMT in the form of MT, further increasing the yield) accompanied by 1 g of polymer (MIP2 or NIP2) in an ultrasonic bath at 50 °C. A corresponding sample without polymer was also prepared. An aliquot of the extract from each of the six samples was passed through a 0.45 μm membrane filter at 5, 10, 15, 20, 30 or 60 min. Each saturated adsorption polymer (20, 30 and 60 min of MIPs) was packed into a glass SPE cartridge and eluted with 20, 10 and 10 mL of water (at 50 °C) to remove impurities and then eluted with 30 mL of water to obtain the target compounds (at 25 °C). Other MIPs and NIPs, eluted with 60 mL of water (at 25 °C) to obtain the target compounds to calculate the dissolution rate of MT from Radix Sophorae tonkinensis.

## 4. Conclusions

The design and synthesis of thermally responsive materials for use in the extraction of MT were demonstrated in this study. An adsorption phase consisting of molecular recognition and thermally responsive elements was developed and evaluated for application using MIP2 as a sorbent material coupled with ultrasonic agitation for the extraction of MT using water as solvent from radix Sophorae tonkinensis. The thermoresponsive MIP displayed different efficiency in clean-up and enrichments using the SPE protocol at different temperatures. The results indicate that the cold water elution of the target compounds after the removal of impurities with hot water were feasible. This approach provides a new method for the extraction of MT in green process. In order to improve the imprinting factor, further functional monomers and polymerization method are currently in progress in our laboratory.
